# Displacement and Isolation: Insights from a Mental Stress Survey of Syrian Refugees in Houston, Texas, USA

**DOI:** 10.3390/ijerph19052547

**Published:** 2022-02-22

**Authors:** Fatin Atrooz, Tzuan A. Chen, Brian Biekman, Ghalya Alrousan, Johanna Bick, Samina Salim

**Affiliations:** 1 Department of Pharmacological and Pharmaceutical Sciences, College of Pharmacy, University of Houston, Houston, TX 77204, USA; fyatrooz@uh.edu (F.A.); galrousa@central.uh.edu (G.A.); 2 Department of Psychological Health and Learning Sciences, College of Education, University of Houston, Houston, TX 77204, USA; tchen3@central.uh.edu; 3 HEALTH Research Institute, University of Houston, Houston, TX 77204, USA; jrbick@uh.edu; 4 Department of Psychology, College of Liberal Arts and Social Sciences, University of Houston, Houston, TX 77204, USA; brian.biekman@times.uh.edu

**Keywords:** Syrian refugees, stress, trauma, displacement, refugee mental health

## Abstract

(1) Background: Syrians are the largest forcibly displaced population in the world. Approximately 20,000 Syrian refugees have resettled in the United States (US) since the civil war in Syria began in 2011, with an estimated 130 families resettling in Houston, Texas. We conducted a pilot study with the objective of examining the physical and mental well-being of the Houston Syrian refugee population. (2) Methods: Online surveys were conducted using psychometrically valid instruments including Afghan Symptom Checklist (ASC), Refugee Post-Migration Stress Scale (RPMSS), Perceived Stress Scale (PSS), and Self-Report Questionnaire (SRQ) (3) Results: According to independent *t*-tests, Syrian refugee females scored higher than males on ASC (37.78 vs. 31.64, *p* = 0.0446), particularly in the subscales of sadness with social withdrawal (28.89 vs. 24.31, *p* = 0.0495), and stress-induced reactivity (6.56 vs. 4.86, *p* = 0.0004). Similarly, females scored higher than males in RPMSS (60.54 vs. 45.15, *p* = 0.0022), including the social strain domain (8.08 vs. 5.18, *p* = 0.0204). In PSS and SRQ, Syrian refugee females reported comparable stress and distress scores as males. (4) Conclusions: Syrian refugee females reported higher stress and distress than males. Displacement from their home country and social strain were the major sources of stress in Syrian refugee females, as indicated in RPMSS.

## 1. Introduction

The Syrian war, which began in 2011, is described as one of the world’s greatest humanitarian crises in modern history. As a result of the political turmoil, war, and destruction, Syrians have been forced to flee, making them the largest forcibly displaced population in the world, amounting to 13 million, including more than 6.7 million internally displaced. The Syrian population fled to several neighboring countries including Turkey, Lebanon, Iraq, Jordan, and Egypt [1]. Turkey hosts the largest number of Syrian refugees amounting to 3.6 million [2]. According to the data from the United Nations High Commissioner for Refugees (UNHCR), an estimated 20,000 Syrian refugees have come to the United States (US) since the civil war in Syria began in 2011. Approximately 130 families have resettled in Houston, Texas. Prior to their resettlement, most Syrian refugees reported experiencing traumatic events including torture of self or loved ones, death of family members or friends, and loss of property, income, and self-sufficiency [3].

The resettlement of refugees in general is a slow process involving temporary crowded, ill-managed, and poorly equipped refugee camps in multiple countries [4,5,6,7]. For Syrian refugees, it has been particularly hard, considering their long journey, often having lived in refugee camps in Jordan, Lebanon, Turkey, Egypt, and Iraq (UNHCR, 2015). Having lived through the traumatic experiences of war and displacement, the Syrian refugees face new stressors upon re-settlement in the US. Although the new environment in the host country, the US, is safe and protected, the new stressors of becoming a minority in a new country, learning a new language, and new social and cultural norms add multiple layers of stress on top of an already stressed and trauma-inflicted life. It is well-known that populations who live in places ravaged from armed conflict, terror, and war often develop posttraumatic stress disorder (PTSD) [3,8,9,10] and other mental health problems [11,12]. Interestingly, resilience following the experience of traumatic events also has been reported in some who have experienced traumatic events of war, conflict, and displacement [13,14,15,16,17].

The present study was designed to explore the mental health and general well-being of Syrian refugees who are resettled in Houston, Texas, US. Experience of PTSD-associated symptoms, aggression, general physical and metabolic health (presence of chronic illnesses like diabetes, hypertension, hypothyroidism, asthma, and irritable bowel syndrome), and sleep status were investigated. We also examined gender differences as women refugees are described as the most vulnerable among the refugee population [18,19,20]. The impact of the new conditions of the host communities of Houston, such as work status and general income, were also explored. Other factors such as age, gender, education, cigarette and hookah smoking habits were included, as they play a significant role in stress reactivity and stress management in this population [20,21].

## 2. Materials and Methods

All communication forms and survey questionnaires utilized in the study were approved by the Institutional Review Board (IRB) Committee (STUDY00002065) for the Protection of Human Subjects, University of Houston (UH), Houston, TX, US.

### 2.1. Subject Recruitment

Upon approval of the study protocol by the UH-IRB Committee, Syrian refugees were recruited to participate in the study through the Houston-based 501 (C) (3) non-profit organization IMPACTs, which facilitates re-settlement of Syrian refugees in the Houston area. The basic criteria of inclusion were exposure to Syrian war and conflict before coming to the US. The refugees might have arrived at another American city but must at present live in the Houston area. The Syrian refugees who agreed to participate in the study were contacted via phone in Arabic. The postdoctoral fellow involved in this study is an Arab American who was responsible for describing study objectives, protocol criteria, and survey instructions to the participants in their native language, following which a secure REDCap survey link was sent via text messages. Participants completed the survey questionnaire either independently or with some guidance via phone by the post-doctoral fellow. Upon survey completion, each participant received a $25 gift card.

### 2.2. Measures

Surveys included a sociodemographic questionnaire with general questions on the demographic and psychosocial circumstances of the respondents including age, gender, education level, socio-economic status, relationship status, and health care access. Questions related to smoking habits and prevalence of chronic diseases (diabetes, hypertension, hypothyroidism, asthma, and irritable bowel syndrome) were included in the participant characteristics section, as these measures are reported to be correlated with stress and mental well-being [21,22,23,24]. Measures of mental health, stress and distress, post-conflict situations, and post-migration stress sources included validated Arabic versions of Self-Reporting Questionnaire (SRQ), Perceived Stress Scale (PSS), Afghan Symptoms Checklist (ASC), and Refugee Post-Migration Stress Scale (RPMSS), respectively.

The SRQ was developed by the World Health Organization by collecting data of patients who contacted primary health care settings for the screening of mental disorder [25]. The questionnaire consists of 24 questions, 20 related to neurotic symptoms, and 4 related to psychotic symptoms. Each of the 24 questions is scored 1 or 0: a score of 1 indicates that the symptom was present during the past month; a score of 0 indicates that it was absent [26]. Different cut-off scores for the SRQ-20 have been selected in different studies, depending on the criteria, culture, and language, but the most prevalent cut-off is considered to be 7. A score of 7 or above indicates the presence of a potential psychological problem. In this study, we used the short form of the SRQ (SRQ-20), which consists of the first 20 non-psychotic items, as this instrument was previously validated in the Arab population [27,28,29]. In our sample, the SRQ-20 also showed high reliability, with a Cronbach’s alpha value of 0.87.

The PSS questionnaire is designed to assess the level of perceived stress experienced over a period of one month [28]. The PSS-14 instrument consists of 7 positive items and 7 negative items rated on a 5-point scale (0 = Never, 1 = Almost Never, 2 = Sometimes, 3 = Fairly Often, 4 = Very Often). Items 4, 5, 6, 7, 9, 10, and 13 are the positively stated items. The scores are calculated by re-versing the scores on the seven positive items, i.e., 0 = 4, 1 = 3, 2 = 2, 3 = 1, and 4 = 0, and then summing across all the 14 items [30]. The Arabic version of the PSS-14 has been previously validated [31]. The PSS-14 showed adequate reliability in our sample, with a Cronbach’s alpha coefficient of PSS of 0.79.

The ASC was developed and used in Kabul, Afghanistan, to identify local indicators of psychological distress in conflict and post-conflict situations [11]. The instrument demonstrated excellent reliability (α = 0.93) and good construct validity. The ASC is a 22-item instrument asking about one’s feelings and experience over a period of 2 weeks. The answers are rated on a 5-point scale (1 = never, 2 = one day each week, 3 = 2–3 days each week, 4 = 4–5 days each week, 5 = every day). The questionnaire consists of three interpretable factors: (1) sadness with social withdrawal and somatic distress, (2) ruminative sadness without social isolation and somatic distress, and (3) stress-induced reactivity, indicated by quarreling, beating one’s children, and nervousness. Among the items are three Dari terms (Dari is the native language spoken by people from Afghanistan) representing Afghan idioms of distress: *jigar khun*, a term describing a form of sadness that includes grief following interpersonal loss; *asabi*, a term for feeling nervous or highly stressed, and *fishar*, or “blood pressure”, which refers to internal agitation or low energy and motivation [11,32]. These terms were translated into Arabic utilizing culturally appropriate terms.

The RPMSS was recently developed and validated among refugees from Syria recently resettled in Sweden [31]. The survey consists of 21 items covering 7 hypothesized domains of post-migration stress: (1) perceived discrimination, (2) lack of host country-specific competencies, (3) material and economic strain, (4) loss of home country, (5) family and home country concerns, (6) social strain, and (7) family conflicts. Each domain has at least 3 items. The answers are rated on a 5-point scale (1 = never, 2 = seldom, 3 = sometimes, 4 = often, 5 = very often). The scores were calculated by adding the scores of all items. The RPMSS showed adequate reliability in our sample, with a Cronbach’s alpha coefficient of 0.92.

### 2.3. Translations

Validated Arabic versions of the PSS, RPMSS, and SRQ were used [28,29,31,33,34]. For ASC, linguistic translation was prepared following linguistic validation guidelines. The linguistic validation of translation consisted of four steps: in the first step, forward translation (English to Arabic) was prepared by a bilingual post-doctoral fellow working on this study. The post-doctoral fellow is a native Arabic speaker and was born and raised on the Syrian–Jordanian border town of Irbid, Jordan. In the second step, backward translation (Arabic to English) was kindly provided by two bilingual faculty members at the University of Houston. In the third step, comparison between back translation version and original questionnaire was conducted by the primary investigator; whenever discrepancies were found between the two versions, the Arabic expressions were adjusted until no further discrepancies were found between the two versions. In the fourth step, the translated version was tested on five Arabic respondents to determine whether the translation was acceptable.

### 2.4. Online Survey

Both versions (Arabic and English) of the survey questions were uploaded on the REDCap platform which enables secure building and management of online surveys. The survey link was sent to the participants via a text message. Upon clicking on the link in the text message, the participants were able to choose the language of survey completion. Some participants preferred the questions be read to them over the phone, so we collected their responses via a phone call interview.

### 2.5. Data Analysis

Data were first examined using descriptive statistics. Sample comparisons between males and females were performed using independent *t*-test or chi-square test for continuous and categorical variables, respectively. Analysis of covariance (ANCOVA) was used to examine the association of gender and the stress scale while controlling for participant age. ANCOVA was conducted separately for each stress scale. The intercorrelation between each stress scale and participant characteristics were examined. Significance was set at *p* < 0.05. All analyses were conducted using SAS software (SAS Institute Inc. Cary, NC, USA, 2014, version 9.4).

## 3. Results

Data were collected from a total of 94 Syrian refugees (38 males, 55 females, and 1 non-binary). The non-binary participant was not included in the analytic sample. Of the 94 participants, 22 completed the questionnaires in English, 72 in Arabic. Some of the young respondents, who completed some college here in the US, chose to answer the survey in English, while most of the respondents whose English language was poor or very limited preferred to complete the Arabic version of the survey.

### 3.1. Demographics

Table 1 depicts the demographic characteristics and social circumstances of the participants overall and by gender. Participant age ranged between 17 and 61 years (Mean = 36.11, SD = 9.85). Overall, 56.52% (N = 52) of this group reported as having education below high school, 25.27% (N = 23) reported working more than 15 h per week, and 80.22% (N = 73) reported as having health insurance. Further, 79.35% (N = 73) were married, 83.52% (N = 76) reported living with a spouse/partner, 60.23% (N = 53) reported a total household income < $10,000, 78.49% (N = 73) and 86.02% (N = 80) reported non-cigarette or Hookah smoking, respectively.

Chi-square tests indicated significant associations between gender and age group (*p* = 0.0313), household income (*p* = 0.0375) and smoking status (*p* < 0.001). Compared with male participants, female participants were more likely to be in the 26–39 years age group (53.70% vs. 26.32%), had higher total household income (49.02% vs. 27.03%), and reported as being non-smokers (98.18% vs. 50%).

### 3.2. Survey Scores and Gender

**Figure 1:**
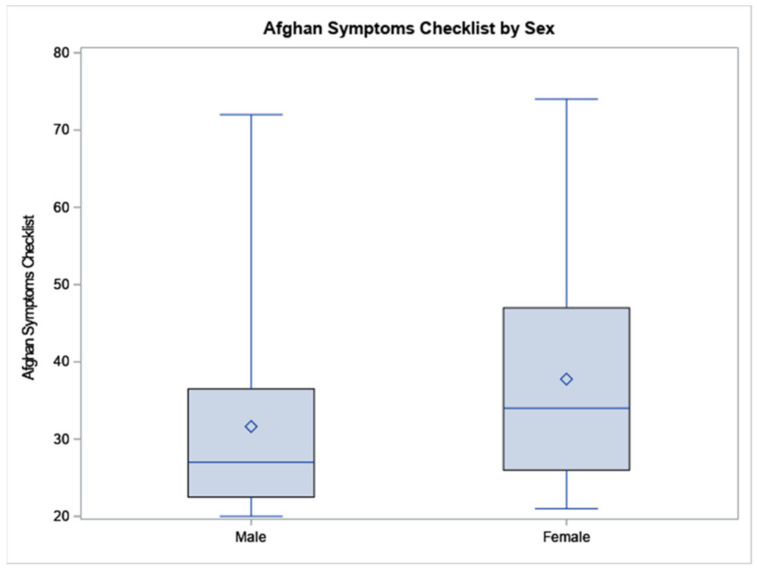
The data are displayed as a box-and-whisker plot. The boxes indicate the intervals between the 25th and the 75th percentiles, the horizontal bars inside the boxes indicate the medians, and ◊ indicates the means. The whiskers indicate the data intervals. Data were analyzed using independent *t*-tests. N = 38 males and 55 females.

**Figure 2:**
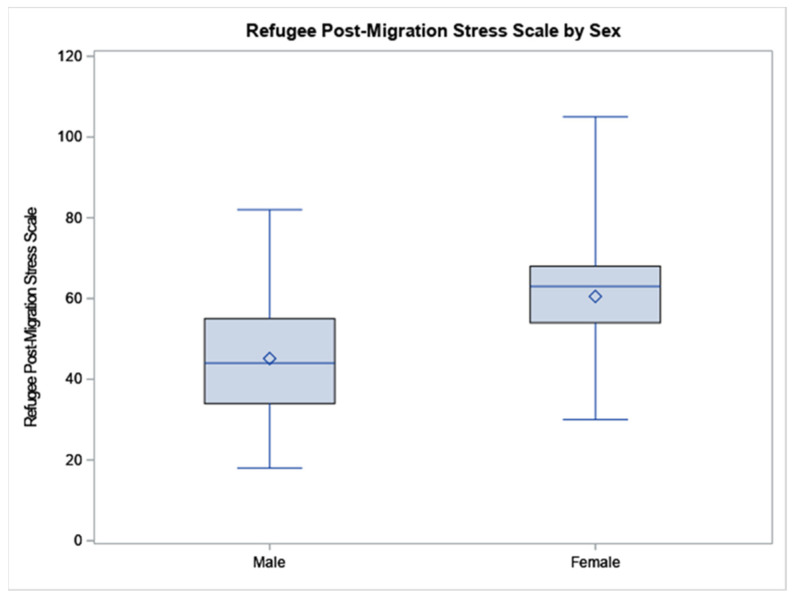
The data are displayed as a box-and-whisker plot. The boxes indicate the intervals between the 25th and the 75th percentiles, the horizontal bars inside the boxes indicate the medians, and ◊ indicates the means. The whiskers indicate the data intervals. Data were analyzed using independent *t*-tests. N = 38 males and 55 females.

According to the independent *t*-tests, Syrian refugee females scored higher than males on ASC (37.78 vs. 31.64, *p* = 0.0446), see Figure 1, particularly in the subscales of sadness with social withdrawal (28.89 vs. 24.31, *p* = 0.0495) and stress-induced reactivity (6.56 vs. 4.86, *p* = 0.0004). Similarly, females scored higher than men in RPMSS (60.54 vs. 45.15, *p* = 0.0022), see Figure 2, including the social strain domain (8.08 vs. 5.18, *p* = 0.0204).

Similar results were yielded when controlling for refugee age in both ASC and RPMSS, except for RPMSS subscale loss of home country and ASC subscale sadness with social withdrawal. The results of ANCOVAs revealed that there were significant gender differences in RPMSS after controlling for age, but not in SRQ and PSS scales. Post hoc analyses showed that females reported significantly higher distress relative to males in ASC (*p* = 0.0469), particularly, in the stress-induced reactivity subscale (*p* = 0.0014). Similarly, in RPMSS, females reported significant higher stress relative to males (*p* = 0.0017), which was evident in the loss of home country domain (*p* = 0.0455) and social strain domain (*p* = 0.0195).

### 3.3. Correlation Analyses 

Correlation analysis indicated that adults who reported a greater number of current health problems were more likely to report more mental health problems, (SRQ: r = 0.369, *p* < 0.001), higher psychological distress (ASC: total: r = 0.289, *p* = 0.006) related to higher sadness with social withdrawal (r = 0.28, *p* = 0.008), and ruminative sadness (r = 0.269, *p* = 0.011) and stress reactivity (r = 0.241, *p* = 0.022) than adults with fewer health concerns.

Socioeconomic and family risk factors were also related to adults’ mental health and stress. Lower income was associated with significantly greater social strain (r = 0.306, *p* = 0.036), poorer general mental health (SRQ: r = 0.249, *p* = 0.021), and higher psychological distress (ASQ: r = 0.277, *p* = 0.010), specifically related to greater sadness with social withdrawal (r = 0.265, *p* = 0.014) and to ruminative sadness (r = 0.3, *p* = 0.005). Adults living in households with a larger number of children were more likely to report concerns about discrimination (RPMSS: r = 0.300, *p* = 0.005) and more family conflict (r = 0.495, *p* = 0.001), than those with fewer children in the home, see Table 2. Variability on the PSS was generally not significantly associated with individual, family, or socio-economic risk scores.

## 4. Discussion

In the present study, Syrian refugee females reported significantly higher mental stress relative to males in RPMSS, which potentially arises from displacement from their home country and from the social strain of resettlement in a culturally novel environment. This seems highly probable, considering previous reports suggesting the prevalence of social strain in refugee women [35,36,37]. Several important factors may have contributed to the social strain: (1) Syrian refugee women expressed their inadequateness in communicating with non-Arabs, (2) the majority of the women were unemployed and did not drive, hence were mostly confined in their homes, (3) doctor’s visits or shopping trips were always chaperoned by a spouse, (4) spoke minimal or no English. Thus, it is obvious that the interaction of refugee women with Americans or individuals from other minorities was very limited. This, combined with the language barrier, adds to the social strain as it reduces the refugee women’s ability to share experiences and burdens with others, increasing the risk of low self-esteem and depression [38,39]. Furthermore, the language barrier is a well-recognized impediment in seeking employment, accessing health care services, and building social connections, thus inhibiting quick integration and adaptation into the host country [19,40]. In addition to language barrier and isolation, the longing for the home country and the loss of cultural belonging through forced migration may be an additional source of strain. Syrian refugee men, on the other hand, reported less stress related to the social strain domain, as they were not confined or isolated as the women. Since men were the breadwinners of the family, and through employment built social interaction, they were more proficient in English than women.

Our data showed that living with a partner was significantly associated with perceived stress and refugee post-migration stress. Previous studies have highlighted the fact that refugee women typically have many children, often live in a joint-family system, and consequently bear extra burden of child care, spouse care, as well as care for the elderly who in most cases have disabilities and/or chronic diseases [41]. Thus, the marital pressures, financial insecurity, and high expectations of the husband collectively create a high-stress environment for Arab women. Relevant to this, marriage has been reported as a mental health risk factor for Iraqi women in a study conducted in Sweden [42]. Iraqi and Syrian women share similar cultural pressures of the patriarchal Arab family patterns [43,44,45]. Correlation analysis also indicated that a number of health issues positively correlated with scores of RPMSS. This is not surprising, considering the intricate cause-and-effect relationship between stress and chronic diseases, such as diabetes, hypertension, and irritable bowel syndrome [46,47]. This is in agreement with previous evidence that suggests that stressful experiences affect body metabolism and predispose an individual to chronic diseases such as diabetes [48], hypertension [49], and irritable bowel syndrome [50,51].

Increased stress in Syrian refugee females, as identified in RPMSS, manifested in ASC symptoms. Syrian refugee women reported a significantly higher overall score in ASC when compared to men. Furthermore, refugee women reported higher scores in the ASC subscale of sadness with social withdrawal and somatic distress and in stress-induced reactivity. Correlation analysis indicated that the number of health issues and family income were positively correlated with all ASC domains. Our data and previous studies suggest that refugee women are at a greater risk of mental health problems, such as depression, anxiety, and PTSD, which increases the risk of psychopathological disorders among this vulnerable group [41,52].

In SRQ, Syrian refugee females and males reported comparable scores, which were below the clinical prevalence cutoff of 7. While significant differences were not obtained between males and females in SRQ, the scores of SRQ for both men and women significantly positively correlated with a number of health issues and also with family income. Perceived stress significantly correlated with living with a partner. The family demands, particularly caring for the children, contributed to the perceived stress. Finally, it seems that RPMSS and ASC are more culturally appropriate instruments for the evaluation of mental distress in Syrian refugees than SRQ or PSS. This is not surprising, considering previous studies where ASC and RPMSS were deemed more appropriate and culturally grounded instruments and particularly regarded as more salient for capturing mental distress in women [32].

## 5. Conclusions

In conclusion, our study has revealed the economic, health, and mental health vulnerabilities of Syrian refugees re-settled in Houston, TX. Our preliminary data suggest that Syrian refugees are highly vulnerable to mental health problems and trauma-related distress symptoms, particularly, female refugees who reported significantly higher distress and greater stress symptoms compared with male refugees. Although high trauma-susceptibility and high resilience have both been reported in refugee women [19,21], Syrian refugee women seem to be a highly vulnerable population [20,52]. Therefore, our recommendations for future interventions include the use of culturally sensitive language-tailored community enrichment programs (English language skills, health educational programs for refugees with low literacy, etc.) which will allow greater assimilation of Syrian refugee women into the American society.

## 6. Limitations

Our study has several limitations. First, the reliance on self-report, as opposed to clinical interview; second, a small sample size focused on one city. Therefore, the results cannot be generalized to other similar populations resettling in other cities. Third, the correlational nature of our observations mitigates cause-and-effect conclusions.

## Figures and Tables

**Table 1:** Participant Characteristics by Gender.

	Total	Male	Female	Statistics	*p*-Value
	(*n* = 93)	(*n* = 38)	(*n* = 55)		
	M (SD)/% [%]				
**Demographics**					
Age	36.11 (9.85)	37.37 (11.15)	35.22 (8.82)	1.03	0.306
Age group				6.9265	0.0313
≤25	17.39 (16)	21.05 (8)	14.81 (8)		
26–39	42.39 (39)	26.32 (10)	53.70 (29)		
≥40	40.22 (37)	52.63 (20)	31.48 (17)		
What is your highest level of education?				3.7302	0.0534
Did not complete high school	56.52 (52)	68.42 (26)	48.15 (26)		
GED/High School and above	43.48 (40)	31.58 (12)	51.85 (28)		
Are you currently employed more than 15 h per week?				0.6552	0.4183
Yes	25.27 (23)	29.73 (11)	22.22 (12)		
No	74.73 (68)	70.27 (26)	77.78 (42)		
What is your current relationship status?				0.0063	0.9366
Married	79.35 (73)	78.95 (30)	79.63 (43)		
All others	20.65 (19)	21.05 (8)	20.37(11)		
Are you currently living with a spouse/partner?				0.1779	0.6732
Yes	83.52 (76)	81.58 (31)	84.91 (45)		
No	16.48 (15)	18.42 (7)	15.09 (8)		
Is your partner or spouse employed for more than 15 h a week?				3.1249	0.0771
Yes	31.51 (23)	20.00 (6)	39.53 (17)		
No	68.49 (50)	80.00 (24)	60.47 (26)		
What is your approximate total household income? Include all sources.				4.3298	0.0375
less than $10,000	60.23 (53)	72.97 (27)	50.98 (26)		
$10,000 or above	39.77 (35)	27.03 (10)	49.02 (25)		
How many children live in your household?	4.2 (2.06)	4.03 (1.95)	4.33 (2.15)	−0.67	0.5068
Do you have medical insurance?				0.0666	0.7964
Yes	80.22 (73)	78.95 (30)	81.13 (43)		
No	19.78 (18)	21.05 (8)	18.87 (10)		
Have you been diagnosed with any of the following? Check all that apply:					
Diabetes mellitus	10.75 (10)	7.89 (3)	12.73 (7)	0.5469	0.519
Hypertension	9.68 (9)	7.89 (3)	10.91 (6)	0.2336	0.733
Hypothroidism	9.78 (9)	5.26 (2)	12.96 (7)	1.4983	0.2978
Asthma	5.38 (5)	5.26 (2)	5.45 (3)	0.0016	1
Irritable bowel syndrome	12.9 (12)	13.16 (5)	12.73 (7)	0.0037	1
Number of Health Issues	0.48 (0.73)	0.39 (0.72)	0.55 (0.74)	−0.98	0.3314
Do you smoke cigarettes?				31.2903	<0.0001
Never	78.49 (73)	50.00 (19)	98.18 (54)		
I was (Quit)	4.3 (4)	10.53 (4)	0 (0)		
Occasionally	4.3 (4)	10.53 (4)	0 (0)		
Less than 10 cigarettes per day	7.53 (7)	15.79 (6)	1.82 (1)		
More than 10 cigarettes per day	5.38 (5)	13.16 (5)	0 (0)		
Do you smoke Hookah?				2.2589	0.7113
Never	86.02 (80)	84.21 (32)	87.27 (58)		
I was (Quit)	1.08 (1)	2.63 (1)	0 (0)		
Occasionally	11.83 (11)	13.16 (5)	10.91 (6)		
Daily	1.08 (1)	0 (0)	1.82 (1)		
**Stress Measurements**					
General Mental Health (possible range: 0–20)	4.37 (4.27)	3.47 (3.81)	5.09 (4.50)	−1.78	0.0791
Perceived Stress Sale (possible range: 0–56)	23.74 (8.72)	23.64 (7.85)	23.87 (9.46)	−0.12	0.9049
Afghan Symptoms (possible range: 20–100)	35.29 (14.18)	31.64 (12.82)	37.78 (14.73)	−2.04	0.0446
Sadness with Social Withdrawal (possible range: 15–75)	27 (10.82)	24.31 (10.01)	28.89 (11.12)	−1.99	0.0495
Fishar Ruminative Sadness (possible range: 2–10)	4.09 (2.29)	3.64 (2.09)	4.36 (2.39)	−1.46	0.1467
Stress-Induced Reactivity (possible range: 4–20)	5.88 (2.53)	4.86 (1.51)	6.56 (2.86)	−3.65	0.0004
Refugee Post-Migration Stress Scale (possible range: 21–105)	54.42 (17.30)	45.15 (17.53)	60.54 (13.91)	−3.23	0.0022
Perceived Discrimination (possible range: 4–20)	6.91 (3.62)	6 (2.77)	7.33 (4.01)	−1.11	0.2721
Lack of Host Country-Specific Competences (possible range: 3–15)	10 (4.01)	9.08 (3.82)	11.05 (3.58)	−1.7	0.0961
Material and Economic Strain (possible range: 3–15)	8.15 (3.73)	7.91 (4.09)	8.82 (3.44)	−0.74	0.46
Loss of Home Country (possible range: 3–15)	11.96 (3.55)	11.09 (2.47)	12.97 (3.04)	−1.88	0.0657
Family and Home Country Concerns (possible range: 2–10)	8.02 (2.71)	7.73 (2.20)	8.62 (2.38)	−1.11	0.2723
Social Strain (possible range: 3–15)	7.15 (3.66)	5.18 (3.40)	8.08 (3.57)	−2.4	0.0204
Family Conflicts (possible range: 3–15)	3.88 (2.29)	4 (1.76)	3.97 (2.56)	0.03	0.9745

**Table 2:** Correlations between health/stress scales and participant characteristics.

		Gender (ref: Male)	Age	Education (ref: Did Not Complete High School)	Employment (ref: No)	Married (ref: All Others)	Living Partner (ref: No)	Partner Employment Status (ref: No)	Family Income (ref: Less than $10,000)	Number of Children Living in Your Household	Health Insurance Status (ref: No)	Number of Health Issues	Smoke Cigarettes (ref: Never)	Hookah Use (ref: Never)
**Self-Reporting Questionnaire**	r	0.186	0.037	−0.100	0.007	0.041	0.123	0.140	0.249	0.004	0.028	0.369	−0.039	0.140
	*p*	0.079	0.733	0.351	0.948	0.700	0.251	0.244	0.021	0.968	0.799	0.000	0.716	0.189
**Perceived Stress Sale**	r	0.013	0.078	−0.157	0.016	0.128	0.288	−0.170	0.205	0.148	−0.045	0.195	0.070	0.164
	*p*	0.905	0.468	0.142	0.880	0.233	0.006	0.157	0.060	0.181	0.676	0.068	0.516	0.126
**Afghan Symptoms**	r	0.212	0.026	−0.030	0.136	0.019	0.071	0.020	0.277	0.160	−0.004	0.289	−0.089	0.086
	*p*	0.045	0.809	0.779	0.205	0.856	0.506	0.866	0.010	0.142	0.970	0.006	0.406	0.421
Sadness with Social Withdrawal	r	0.208	0.029	−0.017	0.124	0.038	0.072	0.042	0.265	0.166	0.024	0.280	−0.091	0.060
	*p*	0.050	0.784	0.874	0.247	0.723	0.504	0.727	0.014	0.129	0.822	0.008	0.392	0.574
Fishar Ruminative Sadness	r	0.155	0.003	−0.085	0.189	0.040	0.082	0.015	0.300	0.104	−0.010	0.269	0.033	0.113
	*p*	0.147	0.981	0.426	0.078	0.713	0.449	0.898	0.005	0.344	0.927	0.011	0.761	0.290
Stress Induced Reactivity	r	0.328	0.016	0.020	0.044	−0.100	0.010	−0.036	0.198	0.153	−0.115	0.241	−0.224	0.151
	*p*	0.002	0.879	0.850	0.684	0.347	0.929	0.762	0.068	0.162	0.288	0.022	0.034	0.155
**Refugee Post-Migration Stress Scale**	r	0.416	0.154	−0.078	−0.049	0.137	0.349	0.196	0.127	0.136	0.100	0.297	−0.044	0.099
	*p*	0.002	0.275	0.585	0.729	0.333	0.012	0.225	0.385	0.350	0.492	0.033	0.757	0.484
Perceived Discrimination	r	0.155	0.048	−0.073	0.209	−0.089	0.151	0.021	0.192	0.439	−0.038	0.265	−0.051	0.007
	*p*	0.272	0.737	0.610	0.138	0.532	0.290	0.899	0.187	0.002	0.795	0.057	0.720	0.963
Lack of Host Country Competences	r	0.233	0.058	−0.186	0.103	0.104	0.191	−0.029	0.159	−0.058	−0.114	0.043	0.105	0.051
	*p*	0.096	0.684	0.191	0.466	0.461	0.179	0.858	0.275	0.691	0.429	0.761	0.460	0.721
Material and Economic Strain	r	0.107	0.074	−0.146	0.054	−0.068	0.064	0.214	0.153	0.030	−0.133	0.127	0.248	−0.004
	*p*	0.460	0.612	0.316	0.711	0.639	0.664	0.191	0.304	0.841	0.368	0.381	0.082	0.979
Loss of Home Country	r	0.262	0.183	−0.222	−0.128	0.346	0.243	0.045	−0.014	−0.025	0.199	0.175	0.174	0.030
	*p*	0.066	0.203	0.125	0.377	0.014	0.093	0.788	0.927	0.866	0.174	0.225	0.226	0.838
Family and Home Country Concerns	*r*	0.158	0.114	−0.267	0.082	0.456	0.402	0.116	0.182	0.051	0.399	0.221	0.159	0.009
	*p*	0.272	0.431	0.063	0.573	0.001	0.004	0.481	0.222	0.732	0.005	0.123	0.271	0.949
Social Strain	r	0.327	0.040	−0.082	0.076	0.046	0.346	0.233	0.306	−0.011	0.173	0.226	0.296	0.162
	*p*	0.020	0.783	0.575	0.598	0.749	0.015	0.153	0.036	0.940	0.241	0.115	0.037	0.260
Family Conflicts	r	−0.005	0.040	−0.037	0.352	−0.339	0.026	0.033	0.225	0.495	−0.152	0.110	−0.034	0.222
	*p*	0.975	0.791	0.808	0.016	0.021	0.867	0.849	0.137	0.001	0.320	0.466	0.825	0.138

## Data Availability

The data presented in this study are available on request from the corresponding author. The data are not publicly available due to privacy.
